# Mobile Health, Information Preferences, and Surrogate Decision-Making Preferences of Family Caregivers of People With Dementia in Rural Hispanic Communities: Cross-Sectional Questionnaire Study

**DOI:** 10.2196/11682

**Published:** 2018-12-10

**Authors:** Bo Xie, Jane Dimmitt Champion, Jung Kwak, Kenneth R Fleischmann

**Affiliations:** 1 School of Nursing The University of Texas at Austin Austin, TX United States; 2 School of Information The University of Texas at Austin Austin, TX United States

**Keywords:** mHealth, dementia, caregivers, rural communities

## Abstract

**Background:**

Mobile health (mHealth) technology holds promise for promoting health education and reducing health disparities and inequalities in underserved populations. However, little research has been done to develop mHealth interventions for family caregivers of people with dementia, particularly those in rural Hispanic communities, who often serve as surrogate decision makers for their relatives with dementia.

**Objective:**

As part of a larger project to develop and test a novel, affordable, and easy-to-use mHealth intervention to deliver individually tailored materials in rural Hispanic communities, in this pilot study, we aimed to examine (1) characteristics of people with dementia and their family caregivers in rural Hispanic communities, (2) caregivers’ preferences for types and amounts of health information and participation in surrogate decision making, and (3) caregivers’ mobile device usage and their desire for receiving information via mobile devices.

**Methods:**

This was a cross-sectional survey. A convenience sample of 50 caregivers of people with dementia was recruited from rural health care facilities in Southwest Texas during 3 weeks of April 2017 to May 2017 via word-of-mouth and flyers posted at the facilities.

**Results:**

More women than men were in the patient group (χ^2^_1_=17.2, *P*<.001) and in the caregiver group (χ^2^_1_=22.2, *P*<.001). More patients were on Medicare and Medicaid; more caregivers had private insurance (*P*<.001 in all cases). Overall, 42% of patients did not have a power of attorney for their health care; 40% did not have a living will or advance directive. Caregivers were interested in receiving all types of information and participating in all types of decisions, although on subscales for diagnosis, treatment, laboratory tests, self-care, and complementary and alternative medicine, their levels of interest for decision-making participation were significantly lower than those for receiving information. On the psychosocial subscale, caregivers’ desire was greater for surrogate decision-making participation than for information. Caregivers did not differ in their interests in information and participation in decision making on the health care provider subscale. All but 1 caregiver (98%) owned a mobile phone and 84% had a smartphone. Two-thirds wanted to receive at least *a little* dementia-related information via a smartphone or tablet. The amount of dementia-related information caregivers wanted to receive via a mobile device was significantly greater for women than for men (*U*=84.50, *P*=.029). Caregivers who owned a tablet were more likely to want to receive dementia-related information via a mobile device than those who did not own a tablet (*U*=152.0, *P*=.006).

**Conclusions:**

Caregivers in rural Hispanic communities were interested in receiving a wide range of information as well as participating in making decisions for their relatives with dementia. There is much need for effective mHealth interventions that can provide information tailored to the needs and preferences of these caregivers.

## Introduction

### Background

Mobile health (mHealth) technology has become an important tool for accessing health information, particularly among ethnic minorities; this new phenomenon presents ample opportunities for health researchers, practitioners, and educators to develop and implement health education interventions to improve health literacy and reduce health disparities and health inequities among ethnic minority groups [1]. mHealth has been used, alone or in combination with a traditional approach, to support health education or self-management for a wide range of health conditions, such as eating disorders [2], multiple sclerosis [3], cardiovascular disease [4], HIV [5], and mental illnesses [6], to name just a few. mHealth interventions have also been tested in a variety of age groups ranging from older adults [7] to pregnant or postpartum women [8] to young adults [9]. Preliminary evidence shows promise for the use of mHealth in chronic disease self-management and for improvements in many physical conditions; however, more systematic research is still needed to generate solid evidence for the efficacy of mHealth-based interventions [10].

Many mHealth interventions have targeted patients, but relatively few have focused on caregivers, and the latter have tended to focus on caregivers of children or youth [11-13]. Our own systematic review suggests that few mHealth interventions have been developed for family caregivers (hereafter *caregivers*) of people with dementia [14], with only a handful of exceptions published within the last few years [15-19].

### The Need to Support Dementia Caregivers’ Decision Making

Dementia has become a major public health concern worldwide. It is estimated that every 3 seconds someone somewhere in the world develops dementia [20]. Nearly 50 million people worldwide were estimated to be living with dementia in 2017, and this number is expected to reach 131.5 million by midcentury [20]. In the United States, Alzheimer disease, which represents the majority of dementia cases, has become the sixth leading cause of death overall and the fifth leading cause of death in older Americans aged 65 years and above [21]. The number of American people living with Alzheimer disease and related dementias (ADRD) is estimated to be 5.7 million in 2018, and this number is expected to increase to 13.8 million in 2050 [21]. The nature of this condition requires extensive care for people with dementia: it is estimated that in 2017, over 16 million informal caregivers in the United States, most of whom were family members, provided 18.4 billion hours of care [21].

Decision making in the treatment and care of people with dementia falls mostly on caregivers, who are expected to make informed decisions in the patient’s best interest. However, caregivers often report being unprepared for their roles and responsibilities, uninformed about treatment options, uncertain about patients’ preferences, and unsupported by professionals in their decision making [22-25]. A major challenge for caregivers is to obtain relevant information about treatment and care options so that they can evaluate the relative merits and risks of each option before making decisions [23,26]. Caring for people with dementia increases risks for caregivers’ mental and physical well-being and deserves much attention [21]. In recognition of the need to support patients and families in making end-of-life (EOL) decisions and to improve EOL care, national projects and federal agencies such as the National Institutes of Health have prioritized advancing current knowledge about EOL decision making and developing innovative decision support interventions for patients with terminal illnesses and their families [27-29]. Given the projected growth of the population with advanced ADRD over the next 50 years [30], the significance of research designed to support caregivers in making EOL care decisions for their relatives will continue to grow.

### Gaps in Existing Interventions for Dementia Caregivers

Interventions supporting caregivers’ decision making are only beginning to emerge; in our recent systematic literature search [14], we found 5 published studies of decision aids for American caregivers in the last 10 years. These decision aids provided caregivers with information about treatment options, but our review identified major knowledge gaps: (1) all study samples were predominately white, (2) existing research has paid little attention to caregivers in rural areas, and (3) existing interventions have included no technology other than audio or video. Thus, no intervention has taken full advantage of recent technological developments to enable the provision of electronic contents tailored to caregivers’ preferences for different types and amounts of information and participation in decision making [14].

These knowledge gaps must be addressed for several reasons. First, for people with dementia and their caregivers of racial or minority backgrounds, there may be special challenges to engaging in advance care planning or in accessing adequate EOL care; the literature has consistently documented cultural differences and disparities at EOL. A systematic review [31] has found that people with dementia from certain ethnic minority groups prefer different EOL treatments and are less likely to have advance directives because of disparities and differences in cultural values. African Americans, for example, are more likely to choose life-sustaining treatment than non-Hispanic whites based on factors such as fear that providers would undertreat, gaps in information and knowledge, and differences in cultural evaluations of the benefits and risks of some care options [31]. The low rate of advance care planning among various ethnic groups (eg, Hispanics, Japanese, Koreans, Chinese, and American Indians) has been attributed to cultural aversion to direct communication about serious illnesses and poor prognosis as well as to preferences for group consensus and the family as a decision-making unit [32]. Another literature review [33] has reported differences in the experiences of caregivers from ethnic minority groups, including higher levels of depression and stress among Hispanic caregivers than among non-Hispanic whites, as well as different coping mechanisms. A meta-analysis found that people with dementia from ethnic minority groups were less able to access health and social services [34].

Second, approximately 15% of the US population, 46 million, lives in rural counties [35]. Rural caregivers face unique challenges [36]. Rural residents tend to be poorer, older, and sicker than their urban counterparts [37,38]. Rural services are often spread over long distances, and the cost of transportation and time often drastically decrease their use [39,40]. Rural nursing homes often lack a diversity of health services or health care professionals for people with dementia [41]. Fewer local health services and providers are available, including palliative care and hospice services [42-44]. Rural caregivers have fewer formal services for support and often rely more on informal services [39] and report greater financial burden [45]. Other unique barriers to the use of formal services include stigma of dementia, lack of privacy, beliefs and attitudes, lack of awareness of services, and less acceptability and accessibility of services [46]. Rural caregivers face different expectations of help and support than urban caregivers do: taking care of a family member with dementia might be seen as a part of life or a family responsibility rather than work, and an inability to provide help for a relative with dementia is more likely to be perceived as abandonment of a relative [45]. Moreover, one consistently identified need of caregivers in rural areas is the need for counseling and mental health services [41]. The coping styles of rural caregivers often differ from those of their urban counterparts, suggesting unique needs [47]. These characteristics and health disparities between urban and rural areas call for effective interventions tailored to the unique needs and circumstances of rural communities and caregivers.

Third, shared decision making and patient-centered care require serious attention to individual preferences [48-50]. However, existing research in this area has examined individual preferences for different types and amounts of health information and decision-making participation mainly from *the health care provider’s perspective* —what *providers* think their patients need to know (typically to ensure compliance). Its focus is typically on a limited range of information and decision making (eg, information and decisions related to treatment). Preferences for other important types of information (eg, how to cope psychosocially) and decision making (eg, choosing which provider to go to) are understudied [51-54]. This trend has continued in interventions involving the use of mHealth technology, with existing interventions showing little consideration for individual preferences for the types and amounts of information received via mHealth. Of the systematic reviews we have examined [55-58], *how often* to receive messages) and 22% accommodated preferences for timing (*when* to receive messages) [56]. Such a trend is unfortunate because meta-analyses provide strong evidence that tailored health behavioral interventions outperform nontailored ones [59-61]; recent developments in mHealth offer unprecedented opportunities for providing tailored health behavioral interventions to hard-to-reach populations [62]. Research is much needed to help caregivers take advantage of new opportunities afforded by mHealth so that they can be better prepared to make informed decisions for their relatives.

### Study Aims and Research Questions

This pilot study was part of a larger study plan to develop and test a novel, affordable, easy-to-use mHealth intervention to deliver individually tailored materials to rural Hispanic communities. We chose to focus on Hispanics because they are the second largest ethnic group after non-Hispanic whites and the fastest growing ethnic minority group in the United States and because they are overlooked in existing intervention studies for caregivers [14]. Our long-term goal is to help caregivers make good use of new technological advancements to be better prepared for the wide range of future care needs and care transitions for their relatives. Toward this end, we conducted our pilot study as a first step to understand community needs and determine the feasibility of the planned larger scale mHealth intervention. Specific aims of the pilot study were to understand (1) the characteristics of people with dementia and their family caregivers in rural Hispanic communities, (2) caregivers’ preferences for different types and amounts of health information and decision-making participation, and (3) caregivers’ mobile device usage and their desire for receiving information via mobile devices.

The primary research questions for this pilot study were as follows:

1. What are the main characteristics of people with dementia and their caregivers in rural communities?
2. What are caregivers’ preferences for overall decision making in the family and for specific types of health information and decision-making participation?
3. What are caregivers’ mobile device usage and desire for receiving information via mobile devices?

## Methods

### Design

This was a cross-sectional survey study.

### Participants

A convenience sample of 50 caregivers was recruited from rural health care facilities in Southwest Texas. These facilities provide health care services, including services for people with dementia, for a 5-county rural area near the US-Mexico border. Participants were recruited during a 3-week period in April 2017 to May 2017 via word-of-mouth and flyers posted at the health care facilities. One of the researchers on the team, a family nurse practitioner who has been practicing in this rural community for over 30 years, identified community stakeholders, obtained permission to access facilities and post flyers for the study, and conducted the participant recruitment and data collection at the facilities. To be eligible, participants had to (1) be aged 18 years or older, (2) be able to read and write in English, and (3) self-identify as a family caregiver or have been caring for a relative with dementia or memory problems by assisting with any activities of daily living (ADL) for at least 2 years. No one refused to participate in the study.

### Procedure

Participants completed a survey instrument on paper while visiting a facility. Completion took approximately 20 to 25 min. Informed consent was obtained before any data collection. Each participant received a US $10 gift card after completing the instrument. The study was approved by the institutional review board of the authors’ institution.

### Materials

The instrument included the following:

- Demographics: 27 items about the patient and 8 items about the caregiver.
- ADL: 6 items, each item scored 1 to 4 with a scoring range of 6 to 24; the higher the score, the more dependent the relative.
- Instrumental Activities of Daily Living: 10 items, each item scored 1 to 4 with a scoring range of 10 to 40; the higher the score, the more dependent the relative;
- Health Information Wants Questionnaire (HIWQ): Preferences for health information and decision-making participation [63-66]; the 21-item HIWQ is a validated, self-administered instrument. It includes 2 parallel scales: the Information Preference Scale (IPS) and the Decision-making Preference Scale (DPS). Each scale contains 7 subscales with parallel items in 7 areas: diagnosis (4 items), treatment (3 items), laboratory tests (3 items), self-care (3 items), complementary and alternative medicine (CAM; 3 items), psychosocial aspects (3 items), and health care providers (2 items). On the IPS, participants indicate how much information they would like to have regarding each of the 7 health-related areas on a 5-point Likert scale (1=none, 2=a little, 3=some, 4=most, and 5=all). On the DPS, participants indicate their preferences for participation in each of the 7 parallel types of decision making on a 5-point Likert scale (1=the doctor alone, 2=mostly the doctor, 3=the doctor and myself equally, 4=mostly myself, and 5=myself alone).
- Technology usage: caregivers’ cell phone and tablet usage and desire for receiving health information via mobile devices; 6 items.

### Data Rescoring and Analysis Strategies

Data were entered into an IBM SPSS file by a research assistant (RA). A second RA independently evaluated the data for accuracy, missing data, and out-of-range values. With guidance from both an experienced biostatistician and the first author, any errors or discrepancies in the data were corrected. Descriptive statistics were used to provide a statistical profile of the sample, reporting frequencies and percentages for categorical data and means and SDs for continuous data. Paired sample *t* test and nonparametric tests (Chi-square and McNemar’s) were used to compare the basic demographic characteristics of the patients and their caregivers. The original subscale scores of the HIWQ were calculated as means across relevant items. Using rescoring strategies that we had used in previous HIWQ studies [63-66], we rescaled the original scores to have a mean of 50 and range from 0 to 100 (100=the strongest desire for information or decision-making participation; 0=no desire). Correlational analyses (Spearman tests) were conducted, and Mann-Whitney tests determined whether there were significant differences between groups (with the dependent variables being at least ordinal).

## Results

### Main Demographic Characteristics of People With Dementia and Their Caregivers in Rural Hispanic Communities

Basic demographic characteristics (age, gender, education, race or ethnicity, and health insurance coverage) of the patients and their caregivers are presented in Table 1. Other key characteristics of the patients as reported by their caregivers are presented in Table 2. Caregivers were significantly younger than the patients: *t*_45_=13.126, *P*<.001. More women than men were in the patient group (χ^2^_1_=17.2, *P*<.001) and in the caregiver group (χ^2^_1_=22.2, *P*<.001). The patient and caregiver groups did not differ in their group compositions in gender, race or ethnicity, or college or no college degree. More patients were on Medicare and Medicaid, whereas more caregivers had private insurance (*P*<.001 in all cases).

### Caregivers’ Preferences for Overall Decision Making in the Family and for Specific Types of Health Information and Decision-Making Participation

Caregivers’ general decision-making patterns in the family and their expectations for who, in general, should make decisions related to their relative’s condition are illustrated in Table 3. Caregivers’ preferences for specific types of health information and decision-making participation are illustrated in Table 4. Caregivers had much interest in all 7 types of information. They also were interested in participating in all 7 types of decision making, although their levels of interest in surrogate decision-making participation were significantly less than their interests in receiving information on 5 of the 7 subscales: diagnosis, treatment, laboratory tests, self-care, and CAM. On the psychosocial subscale, caregivers’ desire for decision-making participation was greater than that for information. Caregivers did not differ in their interests in information and decision-making participation on the health care provider subscale (Table 4). Mann-Whitney tests found no significant difference between women and men, Hispanics and whites, smartphone owners and nonowners, or tablet owners and nonowners in the amounts of specific types of information or decision-making participation they wanted.

**Table 1:** Basic demographic characteristics.

Variable		Patient	Caregiver
**Age (years)**			
	Mean (SD)	78.96 (9.29)	52.62 (13.78)
	Median (range)	81.00 (60-95)	53.00 (27-85)
**Gender, n (%)**			
	Female	39 (78)	41 (82)
**Education, n (%)**			
	8th grade (middle school) or less	24 (48)	4 (8)
	Attended high school	2 (4)	3 (6)
	Completed high school	6 (12)	7 (14)
	Vocational training (after high school)	1 (2)	6 (12)
	Attended college (did not graduate)	3 (6)	12 (24)
	College graduate	10 (20)	15 (30)
	Graduate school	0 (0)	0 (0)
**Race or ethnicity, n (%)**			
	Hispanic or Latino	34 (68)	34 (68)
	White	16 (32)	16 (32)
	Asian	0 (0)	0 (0)
	American Indian or Alaskan native	0 (0)	0 (0)
	Black	0 (0)	0 (0)
	Native Hawaiian or other Pacific Islander	0(0)	0 (0)
**Health insurance coverage, n (%)**			
	Medicare	41 (82)	12 (24)
	Medicaid	21 (42)	3 (6)
	Private insurance	12 (24)	28 (56)
	Veterans	1 (2)	1 (2)
	None	0 (0)	12 (24)

**Table 2:** Other characteristics of the patients.

Other characteristics		Statistics
**Whom the participant is caring for, n (%)**		
	Mother	29 (58)
	Father	6 (12)
	Husband	3 (6)
	Mother-in-law	3 (6)
	Grandmother	2 (4)
	Friend	2 (4)
	Brother	1 (2)
	Cousin	1 (2)
	Wife	1 (2)
**Where relative lives, n (%)**		
	Alone in own home	14 (28)
	In household with the participant	9 (18)
	With another relative	15 (30)
	In a group environment with assistance (eg, an assisted living facility or group home, but not a nursing home)	3 (6)
	Nursing home	9 (18)
**How long has been doing things for relative that he or she used to do for him or herself (month)**		
	Mean (SD)	33.16 (26.67)
	Median (range)	24.00 (2-96)
**Number of other family members or friends (not including participant) provide care routinely**		
	Mean (SD)	2.74 (2.17)
	Median (range)	3.00 (0-7)
**A professional home health person (paid or free) helps to care for relative, n (%)**		
	Yes	16 (32)
**How long relative has been diagnosed with dementia or Alzheimer (month)**		
	Mean (SD)	41.31 (42.16)
	Median (range)	24 (1-183)
**Activities of daily living**		
	Mean (SD)	11.76 (5.79)
	Median (range)	10.00 (6-24)
**Instrumental activities of daily living**		
	Mean (SD)	26.98 (8.95)
	Median (range)	28.00 (11-40)
**Relative has made legal arrangements to have a health care power of attorney, n (%)**		
	Yes	29 (58)
	Participant is the power of attorney	18 (36)
**Relative has a living will or advance directive, n (%)**		
	Yes	30 (60)
	Relative has shared the living will or advance directive with the participant	21 (42)

**Table 3:** General decision-making patterns and expectations.

Decision-making patterns and expectations		Statistics, n (%)
**Within the family, who makes health care decisions for relative **		
	Relative alone	2 (4)
	Mostly relative	3 (6)
	Relative and myself or other family members equally	20 (40)
	Mostly myself or other family members	11 (22)
	Myself or other family members alone	11 (22)
**Who participant thinks should make decisions related to relative’s condition**		
	The health care provider alone	0 (0)
	Mostly the health care provider	3 (6)
	The health care provider and the family equally	30 (60)
	Mostly the family	10 (20)
	The family alone	5 (10)

**Table 4:** Preferences for 7 types of health information and participation in decision making.

Subscale	Information preference, mean (SD)	Decision-making preference, mean (SD)	*t* value *(df*)	*P* value
Diagnosis	68.58 (35.05)	39.32 (25.13)	4.760 (47)	<.001
Treatment	67.01 (38.21)	40.28 (24.75)	4.236 (47)	<.001
Laboratory tests	68.26 (35.60)	30.76 (27.36)	4.924 (47)	<.001
Self-care	67.38 (34.80)	54.97 (23.80)	2.139 (47)	.04
CAM^a^	70.83 (34.55)	47.74 (26.62)	3.109 (47)	.003
Psychosocial	55.56 (36.80)	68.92 (22.26)	−2.255 (47)	.03
Health care providers	60.16 (39.50)	54.69 (25.99)	0.786 (47)	.44

^a^CAM: complementary and alternative medicine.

### Caregivers’ Mobile Device Usage and Desire for Receiving Information via Mobile Devices

Descriptive results are presented in Table 5. Two-thirds of the caregivers wanted to receive at least *a little* dementia-related information via a smartphone or tablet. Mann-Whitney tests found the amount of dementia-related information caregivers wanted to receive via a mobile device was significantly greater for women than for men (*U*=84.50, *P*=.03). Caregivers who owned a tablet were more likely than those who did not own a tablet to want to receive dementia-related information via a mobile device (*U*=152.00, *P*=.006). No significant difference was found between caregivers who owned a smartphone and those who did not, or between Hispanics and whites, in how much dementia-related information they wanted to receive via a mobile device. Spearman tests found no significant correlation between how much dementia-related information caregivers wanted to receive via a mobile device and their age, education, cell phone usage duration, or tablet usage duration.

**Table 5:** Caregivers’ mobile device usage and desire for receiving information via mobile devices.

Caregiver mobile device use		Statistics, n (%)
**Own a cell phone**		
	Yes	49 (98)
	No	1 (2)
**Own a smartphone**		
	Yes	41 (82)
	No	8 (16)
**How long have used a smartphone**		
	Less than 1 year	2 (4)
	At least 1 year but less than 3 years	3 (6)
	At least 3 years but less than 5 years	6 (12)
	At least 5 years but less than 10 years	16 (32)
	At least 10 years	16 (32)
**Own a tablet (eg, Apple iPad)**		
	Yes	31 (62)
	No	18 (36)
**How long have used a tablet**		
	Less than 1 year	7 (14)
	At least 1 year but less than 3 years	3 (6)
	At least 3 years but less than 5 years	8 (16)
	At least 5 years but less than 10 years	14 (28)
	At least 10 years	1 (2)
**How much dementia-related information would like to receive via a smartphone or tablet**		
	None	17 (34)
	A little	2 (4)
	Some	7 (14)
	Most	7 (14)
	All	16 (32)

## Discussion

### Interpreting Our Study Participants’ Basic Characteristics

This pilot study was part of a larger project to develop and test a novel, affordable, easy-to-use mHealth intervention to deliver individually tailored information to aid caregivers in rural Hispanic communities to make informed decisions for their relatives suffering from dementia. Our long-term goal is to help caregivers take advantage of new technological advancements to prepare for the wide range of future care needs and transitions for their relatives. This study was a first step taken to understand community characteristics and preferences and determine the feasibility of the larger mHealth intervention. We chose to focus on rural Hispanic communities because Hispanics are the second largest ethnic group and the fastest growing ethnic minority group in the United States and because rural communities face unique challenges [37-47]. Hispanics residing in rural areas are at double jeopardy in getting proper health care and services. Existing interventions for caregivers have largely overlooked the special needs and preferences of rural Hispanic residents [14]. More than two-thirds of the patients and caregivers in our study sample were Hispanic and the others were white. The sample contained no patients or caregivers of other racial or ethnic groups. The sample’s race or ethnicity approximately reflects that of the population in the County where 71% of the population is Hispanic, with a few residents belonging to other ethnic minority groups [67].

Census data also show that 15% of people aged above 25 years in this rural Southwest Texas County had college degrees or higher [67]. In our sample, 20% (10/50) of the patients and 30% (15/50) of the caregivers had college degrees. These higher percentages for college education may have been due, at least in part, to the inclusion criterion that participants be able to read and write in English. The majority of the patients (78%; 39/50) were women, with over half of the patients (58%; 29/50) reported as mothers of the caregivers who completed the survey instruments. The majority of the caregivers (82%; 41/50) were women as well. National data suggest that 60% of caregivers (including but not limited to caregivers of dementia patients) in the United States are female [68], almost two-thirds of people with Alzheimer disease in the United States are women, and approximately two-thirds of Alzheimer disease’s caregivers in the United States are also women [21]. It appears that our study sample consisted of even higher percentages of female patients and female caregivers than the national data suggest. These differences in gender composition might be because of our small sample size, such that small differences in numbers could turn into rather large differences in percentages. However, they might also reflect characteristics of the rural Hispanic communities we studied (Hispanic women in rural areas might be even more likely than the general population to be caregivers of people with dementia). Future research should be conducted with larger, more representative samples.

On average, the mean of other family members or friends (not including the research participants themselves) providing care routinely was 2.74 (range 0-7), with a median of 3. Meanwhile, although the majority (76%; 38/50) of the patients lived in a home environment (alone or with a family member), over two-thirds (68%; 34/50) of the patients did not have a professional home health person, whether paid or free, to aid the family in caring for the patient. Together, these findings show a heavy reliance on informal care and an underutilization of formal care for people with dementia in rural Hispanic communities. Our findings suggest that patients and caregivers in these communities face unique challenges in accessing formal health and social services because of, as reported in the literature, their ethnic minority background and residence in rural areas [34,39-44]. Heavy reliance on informal care will likely become an even more serious challenge in the future. As the population ages, increasing numbers of older adults with dementia and/or other conditions will require care so that reliance on informal caregivers will not be sustainable in the long run [69]. Technological developments such as mHealth may be particularly promising for shifting dependency away from informal caregivers while meeting the care needs of the aging population.

All patients had at least some form of health insurance. The vast majority of the patients (82%; 41/50) were on Medicare and a large portion (42%; 21/50) was on Medicaid. The high percentage of patients on Medicaid is not surprising, because 25% of residents in this rural Texas County live in poverty [62]. A majority of caregivers had private health insurance (56%; 28/50), whereas 24% (12/50) of caregivers had no health insurance at all. These findings suggest additional challenges unique to patients and caregivers in rural ethnic minority communities, that is, they tend to be poorer, older, and sicker than their urban counterparts [37,38]. Notably, 42% (21/50) of the patients did not have a power of attorney for their health care and 40% (20/50) did not have a living will or advance directive. These findings also suggest unique challenges that rural Hispanic communities face, and they too are in line with those in the literature; ethnic minority groups, including Hispanics, have low rates of advance care planning [31,32]. These findings illustrate the need for effective interventions in rural Hispanic communities.

The majority (62%; 31/50) of health care decisions in the family were made with some form of shared decision making between the patient and family members; 10% (5/50) of decisions were made by the patient alone or mostly by the patient. However, 22% (11/50) of decisions were made by family members alone without involving the patient. Beyond the family, the majority (60%; 30/50) of caregivers felt that the health care provider and the family should play equal roles in making decisions. Another 30% (15/50) felt that the family mostly or the family alone should make all decisions. No caregiver thought that the health care provider should make decisions alone. In terms of caregivers’ preferences for specific types of health information and decision-making participation, our data showed that caregivers were interested in a broad range of health information and decision-making participation, although their levels of interest varied across the 7 subscales and between information and decision-making preferences. Caregivers had a strong desire for all 7 types of information: on a 1 to 100 scale, where 1 indicated the least amount of information wanted and 100 the greatest amount of information wanted, participants scored from 55 to 71 on the 7 types of information wanted. They were also interested in all 7 types of decision-making participation, although their interest in decision-making participation was significantly lower than their interest in information on 5 of the 7 subscales (diagnosis, treatment, laboratory tests, self-care, and CAM). On the psychosocial subscale, however, caregivers’ desire for decision-making participation was greater than that for information. On the health care provider subscale, no significant difference was found between caregivers’ interests in information and decision-making participation. These findings are similar to those of earlier studies using the HIWQ in different samples [66,70], suggesting generalizability across populations in individual preferences for health information and decision-making participation.

Our data show that all but 1 (98%) of the participants had a mobile phone; however, 16% (8/50) lacked a smartphone. This is consistent with national data: as of January 2018, 95% of the US population had a mobile phone, whereas 17% lacked a smartphone [71]. Although the percentages of people with mobile phones in urban, suburban, and rural areas were approximately the same, rural areas had a greater percentage of people who had nonsmart mobile phone devices (26%) than urban areas (13%) [71]. In addition, those with less than high school education and those who made less than $30,000 a year had higher percentages of nonsmartphone use (33% and 25%, respectively) than the national average [71]. These findings further suggest unique challenges that rural communities often face (eg, poverty and lack of formal education), and they have implications for interventions targeting caregivers in rural areas. Specifically, although smartphones have many advantages over nonsmartphones, mHealth interventions that do not require smartphones (eg, short message service [SMS] text messages supported by all mobile phone devices, smart or nonsmart) may be the best way to reach the most caregivers in rural areas, particularly those who cannot afford smartphones and associated data plans.

### Caregivers’ Mobile Health Preferences

In terms of how much dementia-related information caregivers would like to receive via a mobile device, two-thirds of the caregivers went for an extreme: 34% (17/50) preferred to receive *no* information, whereas another 32% (16/50) preferred to receive *all* information via a mobile device. Caregivers’ age, education, race or ethnicity, smartphone ownership, cell phone usage duration, or tablet usage duration did not seem to have a relationship with how much dementia-related information caregivers wanted to receive via a mobile device. Only 2 predicative variables were found. Women wanted to receive more dementia-related information via a mobile device than men. This is not surprising; research has consistently shown that women are more interested than men in obtaining health-related information [72-74]. Moreover, caregivers who owned a tablet wanted to receive more dementia-related information via a mobile device than those who did not own a tablet. This finding is particularly interesting given that no relationship was found between the other mobile device-related variables (smartphone ownership, cell phone usage duration, and tablet usage duration) and the amount of information caregivers wanted to receive via a mobile device.

### Limitations and Future Directions

Due to limited resources, we were able to use only an English instrument; thus, our sample included only caregivers fluent in English and bilingual in English and Spanish. As such, the findings of this study might not be generalized to caregivers not fluent in English. The small sample might also limit the findings’ generalizability. Future research should be conducted with larger and more representative samples. This pilot study focused specifically on mHealth and did not include interventions that were internet-based and that relied largely on computers (for a systematic review, see the study by Hopwood et al [75]). We chose to focus on mHealth mainly because those who live in rural Hispanic communities are more likely to have less formal education and higher levels of poverty and they might be more likely to use mobile devices than computers on a daily basis. However, it might be interesting to explore in future research whether or how mobile device and internet-based interventions might be perceived and used differently and/or similarly by rural caregivers.

### Conclusions

This pilot study generated preliminary data about key characteristics of people with dementia and their family caregivers in rural Hispanic communities, including caregivers’ preferences for different types and amounts of health information and decision-making participation and the needs of rural caregivers for mHealth-based interventions tailored to their unique circumstances. In particular, our data show that 42% (21/50) of the patients did not have a power of attorney for their health care and 40% (20/50) did not have a living will or advance directive. These findings illustrate the need for effective interventions to improve the rates of having a power of attorney and a living will or advance directive in rural Hispanic communities. Compared with the national data, our study found an even higher percentage of female caregivers, perhaps because Hispanic women in rural areas are even more likely than the general population to be caring for their families. Caregivers, women or men, were interested in a broad range of health information and decision-making participation; women, compared with their male counterparts, wanted to have even more dementia-related information via a mobile device. Together, these findings support a need for mHealth interventions that can provide relevant information for caregivers in rural Hispanic communities.

However, in developing mHealth interventions for these caregivers, it is important to bear in mind that although almost all caregivers in our study sample had a mobile phone, 16% lacked a smartphone. This is consistent with the findings for national samples. Thus, mHealth interventions that do not require smartphones (eg, SMS text messages supported by all mobile phone devices, smart or nonsmart) may be the best way to reach the most caregivers in rural areas, particularly those who cannot afford smartphones and associated data plans. Furthermore, caregivers’ levels of interest in dementia-related information and decision-making participation varied across the 7 subscales. Thus, mHealth interventions, smartphone-based or not, should strive to provide information tailored to individual caregivers’ specific preferences (eg, providing more self-care related information to caregivers who want more of such information, whereas providing more CAM-related information to those who want more CAM-related information).

The findings of this pilot study have implications for dementia research, practice, and policy making. Our study of the characteristics of people with dementia and their family caregivers in rural areas, especially those in racial or ethnical minority groups, supports a patient- and family-centered approach to address the significant need for interventions sensitive to underserved populations’ unique situations. Affordable and easy-to-use mHealth interventions can help caregivers obtain desired health information to make informed decisions, even if they have limited technology experience and/or cannot afford the cost of smartphones and services. Such interventions should have a long-term and broad impact on EOL care for people with dementia and their caregivers in the rapidly evolving mHealth era.

## Abbreviations

ADL: activities of daily living
ADRD: Alzheimer disease and related dementias
CAM: complementary and alternative medicine
DPS: decision-making preference scale
EOL: end-of-life
GRA: graduate research assistant
HIWQ: Health Information Wants Questionnaire
IPS: Information Preference Scale
SMS: short message service
PI: principal investigator
mHealth: mobile health
RA: research assistant
